# Invasive Community-Acquired Methicillin-Resistant Staphylococcus aureus With Aortic Aneurysm in a 10-Year-Old Patient: A Case Report

**DOI:** 10.7759/cureus.62712

**Published:** 2024-06-19

**Authors:** Ngozi Onyishi

**Affiliations:** 1 Pediatrics, Driscoll Children's Hospital, Corpus Christi, USA

**Keywords:** abscess, necrotizing pneumonia, aneurysm, sepsis, community acquired mrsa

## Abstract

The clinical presentation of disseminated community-acquired Methicillin-resistant *Staphylococcus aureus* (MRSA) in young pediatric patients without a known predisposing risk factor poses a diagnostic dilemma due to its non-specific clinical symptoms. This can lead to delayed initiation of appropriate antibiotics and surgical interventions to achieve a favorable outcome and prevent mortality. Appropriate imaging with good clinical judgment is required in the management of this infection. Outpatient surveillance for subacute and chronic complications is required for a good long-term prognosis.

Few reported cases of disseminated infections with aortic aneurysm exist in the literature. We report a case of a child without predisposing risk factors managed for community-acquired MRSA sepsis, acute respiratory distress syndrome (ARDS), multiple abscesses, osteomyelitis, and necrotizing pneumonia with a post-discharge unruptured aortic aneurysm. This case emphasizes the importance of post-discharge monitoring even in patients with favorable outcomes.

## Introduction

*Staphylococcus aureus* has been a major cause of morbidity and mortality in the pre and post-antiseptic eras. In 1880, Alexander Ogston discovered the leading cause of post-surgical suppuration and proposed that abscesses were caused by micrococci, which he named Staphylococcus in 1882 [1]. By 1884, Anton J. Rosenbach identified two strains of the organism: *Staphylococcus aureus and Staphylococcus epidermidis *[1]. As epidemiological shift occurs, the genetic shift of antibiotic resistance grows, causing a significant dilemma in the medical field. This organism is either hospital-acquired or community-acquired and has different bacteriological and clinical properties [2]. The staphylococcal cassette chromosome mec (SCC mec) type IV or type V are the two genes responsible for methicillin resistance [2].

Community-associated methicillin-resistant *Staphylococcus aureus* (CA-MRSA) can cause disseminated infections in multiple systems: endocarditis, psoas abscess, epidural abscess, and osteomyelitis, with mortality reaching 30% [3]. CA-MRSA can cause severe necrotizing pneumonia in young, immunocompetent patients [4]. The incidence of invasive CA-MRSA infections increased significantly by 54.5% between 2005 and 2010 [5]. A study done in 2013 among the US pediatric population showed that 42% of MRSA cases were community-associated (CA), 35% were hospital-onset, and 23% were healthcare-associated community-onset [5]. Few pieces of literature on pediatric patients with CA-MRSA septicemia, pneumonia, and osseous involvement have reported favorable outcomes; most reported fatal or poor outcomes [2,6-8]. This case report examines a child with CA-MRSA sepsis, acute respiratory distress syndrome (ARDS), multiple abscesses, osteomyelitis, and necrotizing pneumonia with a post-discharge unruptured aortic aneurysm.

## Case presentation

A 10-year-old female from South Texas was transferred to our hospital from a referral emergency room in respiratory distress. The patient was in her usual state of health until seven days before her presentation while in Mexico, when she had a fall on her right hip. She had an X-ray at that time, which was reassuring, and was sent home. She developed fever (temperature unknown), body and leg swelling, with gait abnormalities. A day before her presentation, she developed respiratory difficulty and anasarca, for which she was taken to a hospital in Mexico, where she was diagnosed with pneumonia, acute kidney injury (AKI), and thrombocytopenia. Due to her acuity, she was transferred to the referral hospital in the US, where she had signs of pneumonia with electrolyte imbalances. A decision was made to transfer her to our hospital for higher care. There was no prior medical or surgical history. She lived in South Texas but occasionally traveled to Mexico to visit family.

On admission, she was toxic-looking, agitated, confused, and in respiratory distress. Her Glasgow Coma Scale (GCS) was 14 and she had abnormal vital signs with a heart rate of 155 beats per minute (b/minutes), respiratory rate of 38 breaths per minute, temperature of 36.9 ^o^C (degrees centigrade), and blood pressure of 94/60 millimeter mercury (mmHg). She had chapped lips and dry buccal mucosa, coarse breath sounds on all lung zones, and normal first and second heart sounds with no murmur. Her abdomen was soft and nontender with no enlarged liver or spleen. She had bilateral knee swelling with pitting pedal edema and scattered petechiae on her lower extremities bilaterally.

Investigations

The patient had a significantly elevated white blood cell, anemia, and thrombocytopenia. She had hyponatremia, hypocalcemia, hyperkalemia, and metabolic acid-base imbalance. She had high C-reactive protein and erythrocyte sedimentation rates (Table 1).

**Table 1:** Laboratory investigations milliliter (ml), percent (%), gram per deciliter (gm/dl), microliter (ul), milligrams per deciliter(mg/dl), grams per deciliter(g/dl), millimoles per liter (mmol/L), units per liter (u/L), international units per liter (IU/L), millimeters of Mercury (mmHg), partial pressure of carbon dioxide (pCO2), partial pressure of oxygen (pO2), picogram per milliliter (pg/ml), micrograms per millimeter (ug/ml), millimeter per hour (mm/hr)

Parameter	Patient’s value	Normal reference value
White blood cells	23600	3500 -10500 per ml
Neutrophils	30	35-75%
Lymphocytes	4	24-48%
Band	29	0-10%
Hemoglobin	10.6	11.8-14.8 gm/dL
Hematocrit	31.8	33.0-44.0 %
Platelet	11000	150,000-450,000 per ul
Glucose	85	75-100 mg/dl
BUN (blood urea nitrogen)	69	6-17 mg/dl
Creatinine	0.76	0.60-1.00 mg/dl
Sodium	129	132-144 mmol/L
Potassium	5.2	3.3-4.7 mmol/L
Calcium	7.5	8.8-10.5 mg/dl
Ionized calcium	0.88	1.22-1.37 mmol/L
Chloride	97	97-107 mmol/L
Carbon dioxide	16	16-25 mmol/l
Total protein	4.9	6.4-8.2 g/dl
Albumin	1.7	3.8-5.6g/dl
Total bilirubin	1.8	0.1-1.4 mg/dl
Aspartate aminotransferase	227	10-47 u/L
Alanine aminotransferase	122	10-47 u/L
Alkaline phosphatase	83	169-657 u/L
Calculated osmolality	287	282-300
Anion gap	16	8-16
pH, venous	7.38	7.31-7.41
PCO2, venous	22	41-51 mmHg
PO2, venous	171	24-48 mmHg
Bicarbonate, venous	12.9	23-28 mmol/L
Lactate	6.9	0.9-1.7 mmol/L
Lactate dehydrogenase	1005	129-222 IU/L
Brain natriuretic peptide	348	2-100 pg/ml
Troponin I	100.5	20-100 pg/ml
Creatinine phosphokinase	3515	25-177 U/L
Prothrombin time	16.7	12.4-14.9 seconds
PTT (partial thromboplastin time)	29	21-39 seconds
D-dimer, quantitative	7.42	<=0.50ug/ml
Fibrinogen	601	158-396 mg/dl
C-reactive protein	28.8	<0.8 mg/dl
Erythrocyte sedimentation rate	64	0-20 mm/hr

**Figure 1:**
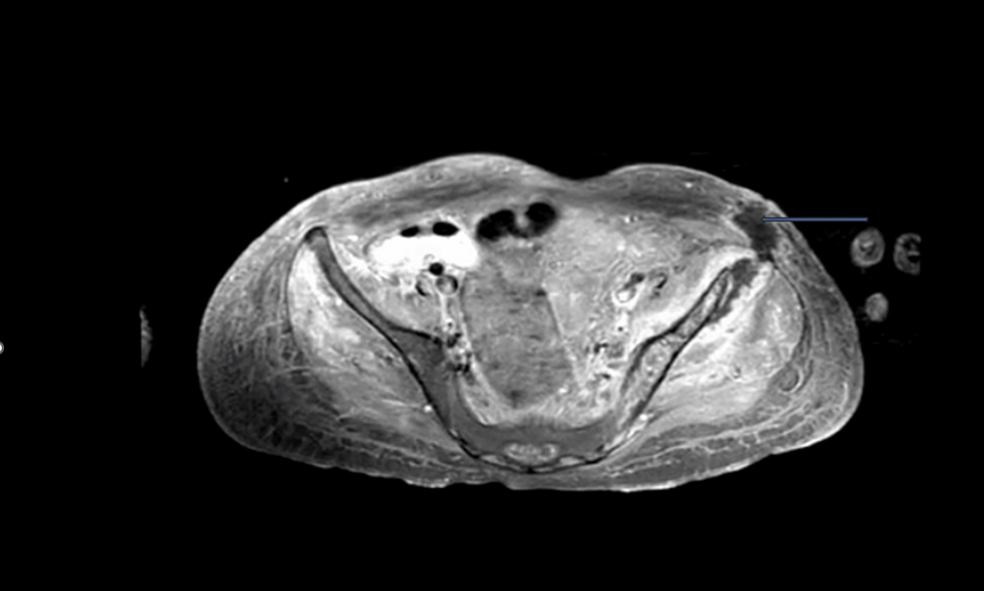
Magnetic resonance imaging of the pelvis The blue arrow shows the left gluteal abscess.

Chest X-ray showed multifocal patchy opacification throughout both lungs, and X-ray of the left hip and ankle showed diffuse soft tissue swelling with no bone abnormality. Ultrasound of the thyroid gland showed multiple sub-centimeter space-occupying lesions within the thyroid measuring up to 0.8 centimeters in dimension within the mid-left lobe that was not drainable. Ultrasound of the jugular veins bilaterally showed no thrombus within the veins. Abdominal ultrasound revealed hepatomegaly with findings suggestive of hepatitis. It also demonstrated gallbladder sludge with gallbladder wall thickening, trace pericholecystic fluid, and subjectively increased renal parenchymal echogenicity. No inferior vena cava thrombus was identified. Venous Doppler ultrasound of the legs showed no thrombus. Ultrasound of both knees showed no effusion while the elbow joints showed trace effusion and diffuse subcutaneous edema suggestive of cellulitis of the upper limb. A nuclear medicine bone scan showed no definitive osseous uptake to suggest osteomyelitis. Magnetic resonance imaging (MRI) of the brain with and without contrast showed no intracranial dissemination of infection or intracranial lesion. MRI of the pelvis with and without contrast showed osteomyelitis of the left ileum with concurrent multifocal abscess formation. The largest focal fluid collection was seen within the left gluteus minimus, measuring 2.8 cm x 1.8 cm x 6.3 cm in dimension. Bilateral small hip joint effusions with associated synovial enhancement and extensive pelvic muscular, fascial, and subcutaneous edema were seen (Figure 1).

MRI spine with and without contrast showed no evidence of vertebral body osteomyelitis or discitis. No enhancing spinal cord lesions or intra-spinal fluid collections were identified. A 1.8-centimeter abscess was suspected within the left paraspinal musculature at the level of S2.

**Figure 2:**
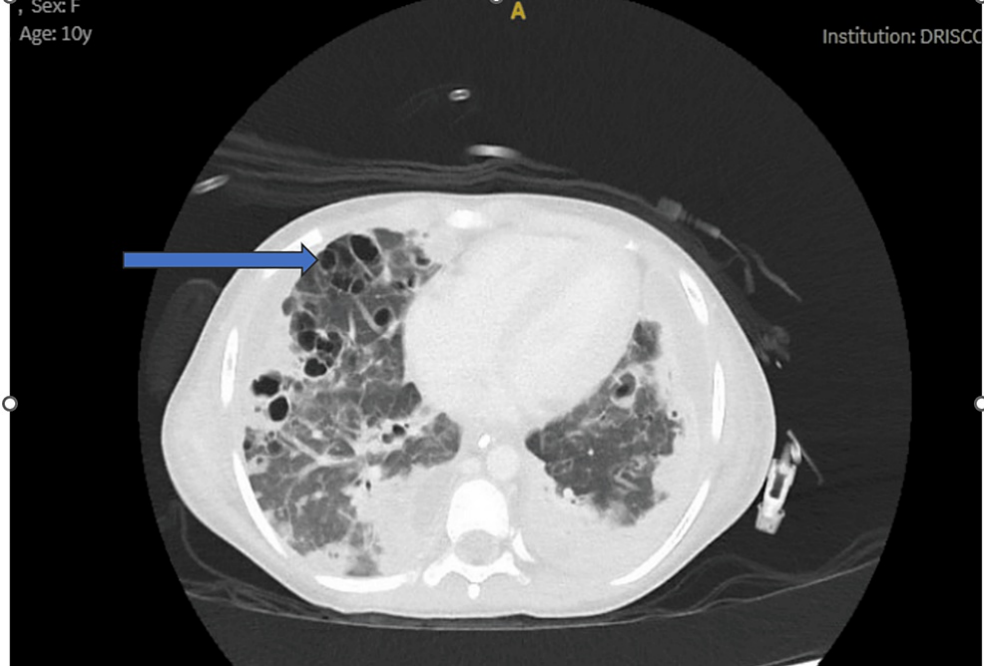
Computed tomography of the chest on admission The blue arrow shows cavitary changes in the right lung.

Computed tomography (CT) of soft tissue neck and chest with contrast showed no acute fracture or focally destructive osseous lesions. A peripheral enhancing retropharyngeal collection was seen, which was concerning for a retropharyngeal abscess. Patchy areas of nonspecific low density within the thyroid measuring up to 8 millimeters (mm) were also identified. Additionally, patchy consolidative and cavitary lesions were found throughout both lungs, possibly related to direct parenchymal infection and/or secondary to infection (Figure 2).

Computed tomography of the abdomen and pelvis with contrast revealed hepatomegaly with heterogeneous opacification, gallbladder edema, and moderate volume ascites with extensive stranding involving the subcutaneous and muscular/fascial components. Echocardiography showed the presence of a small mobile vegetation adherent to the anterolateral papillary muscle. There was normal left ventricular ejection fraction and biventricular function. There was no significant valvular insufficiency, stenosis, or pericardial effusion. An electrocardiogram (EKG) showed sinus tachycardia, left atrial enlargement, and nonspecific ST depression. Blood, respiratory, wound, and joint fluid cultures all grew MRSA. Urine culture also revealed 10,000-25,000 colony-forming units/milliliter (CFU/ml) of MRSA. In addition, the pneumonia polymerase chain reactive (PCR) test revealed methicillin-resistant* Staphylococcus aureus*, *Haemophilus influenzae* type b, and Rhino enterovirus. The Karius test showed the MRSA staphylococcal cassette chromosome (SCC) mec gene. She had low CD4 positive T-cells of 342 cells per microliter and natural killer cells, which was most likely due to systemic steroid use at the time of the test. Repeat flow cytometry done when the patient was off systemic steroids showed improved CD4-positive T-cells of 565 cells per microliter. The B-cell count was initially normal, but with the repeat flow cytometry test, it was low. There was no clinical concern regarding the low B-cell count when reviewed by an immunologist.

Diagnosis

The diagnosis included CA-MRSA, severe sepsis with pneumonia, endocarditis, osteomyelitis of the left ileum, bilateral septic arthritis, bilateral knee arthritis, thyroid abscesses, retropharyngeal abscess, and one paraspinal abscess.

Treatment

She received broad-spectrum antibiotics on admission but was later transitioned to only vancomycin and clindamycin for MRSA coverage. Vancomycin was given for 28 days and discontinued while clindamycin was given for 47 days before she was transitioned to linezolid. The patient was advised to be on linezolid until CRP normalizes.

Outcome and follow-up

**Figure 3:**
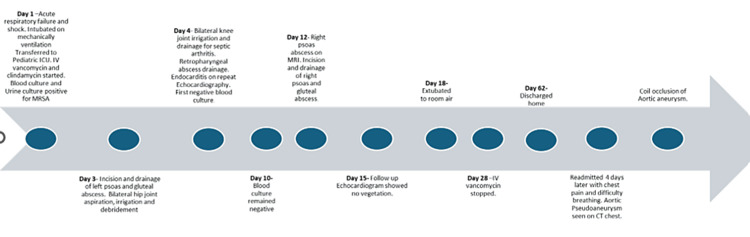
Timeline of admission Intensive care unit (ICU), intravenous (IV)

She was admitted to the pediatric intensive care unit where she was managed for septic shock and acute respiratory failure (Figure 3). She had bilateral hip and knee irrigation, drainage, and debridement (Figure 3). The fever curve improved with down-trending inflammatory markers after these procedures.

On day 12, the patient had unstable blood pressure (BP) with low diastolic BP, requiring an increase in vasopressor use, fever spikes, and up-trending WBC counts with inflammatory markers. Repeat CT of the abdomen/pelvis showed a psoas abscess on the right, and this was confirmed with an MRI of the pelvis.

**Figure 4:**
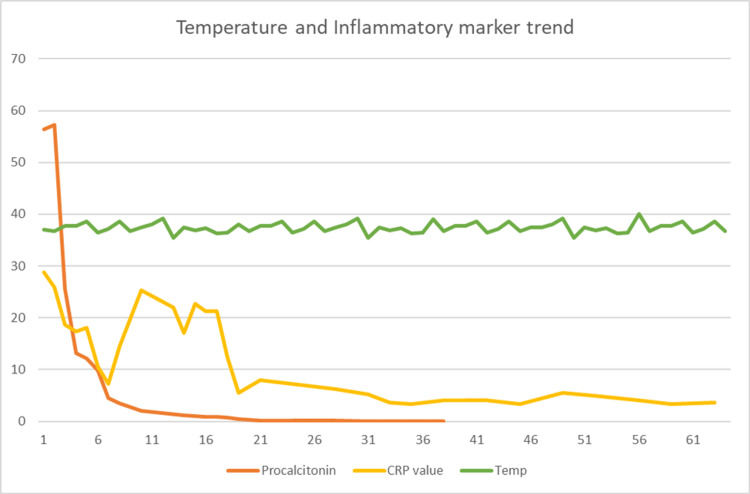
Inflammatory markers and temperature trend on first admission The X-axis is the days of admission and the Y-axis is C-reactive protein, procalcitonin, and temperature values on admission. The graph shows a downward trend of CRP, procalcitonin, and temperature. There was an intermittent spike in temperatures and CRP, however, procalcitonin remained stable. CRP: C-reactive protein in mg/dl (milligram per deciliter); Temp: Temperature in degrees centigrade; Procalcitonin in ng/dl (nanograms per milliliter)

She was taken back to the operating room, where she had Irrigation and debridement of the left iliac crest, drainage of gluteal musculature abscess, and psoas abscess (Figure 3). Procalcitonin and CRP had intermittent spikes but downtrended after the second incision and drainage of the hip abscess (Figure 4 and Table 2).

**Table 2:** Inflammatory markers and temperature trend on first admission The table shows the values of CRP, procalcitonin, and temperature on admission. CRP: C-reactive protein in mg/dl (milligram per deciliter); Temp: temperature in degrees centigrade; Procalcitonin in ng/dl (nanograms per milliliter)

Days	Procalcitonin (ng/ml)	CRP (mg/dl)	Temperature
1	56.41	28.8	37.0°C
2	57.27	25.9	36.7°C
3	25.56	18.7	37.8°C
4	13.17	17.4	37.8°C
5	12.15	18.1	38.6°C
6	9.81	10.5	36.4°C
7	4.49	7.3	37.2°C
8	3.51	14.5	38.6°C
9			36.8°C
10	2.03	25.3	37.5°C
11			38.1°C
12			39.2°C
13		22	35.5°C
14	1.19	17.1	37.4°C
15	0.99	22.7	36.9°C
16	0.82	21.3	37.3°C
17	0.91	21.2	36.3°C
18	0.79	12.4	36.4°C
19	0.43	5.5	38.0°C
20			36.7°C
21	0.19	7.9	37.8°C
22			37.8°C
23			38.6°C
24	0.22		36.4°C
25			37.2°C
26			38.6°C
27			36.8°C
28	0.13	6.3	37.5°C
29			38.1°C
30			39.2°C
31	0.09	5.2	35.5°C
32			37.4°C
33		3.7	36.9°C
34			37.3°C
35	0.07	3.3	36.3°C
36			36.4°C
37			39.0°C
38	0.05	4	36.7°C
39			37.8°C
40		4	37.8°C
41			38.6°C
42		4	36.4°C
43			37.2°C
44			38.6°C
45		3.3	36.8°C
46			37.5°C
47			37.5°C
48			38.1°C
49		5.5	39.2°C
50			35.5°C
51			37.4°C
52		4.9	36.9°C
53			37.3°C
54			36.3°C
55			36.4°C
56		4	40.0°C
57			36.7°C
58			37.8°C
59		3.3	37.8°C
60			38.6°C
61			36.4°C
62			37.2°C
63		3.6	38.6°C
64			36.8°C

Our patient had necrotizing pneumonia on admission, which was shown on imaging as multifocal pulmonary opacification with evidence of cavitation. After about six weeks of admission, repeat imaging showed that the lungs were better aerated. The right pyelonephritis and hepatomegaly improved after six weeks of admission compared to the prior imaging on the first week of admission. Blood culture and urine culture on admission showed MRSA. However, subsequent blood cultures obtained periodically during treatment remained negative (Figure 3).

WBC also decreased with periodic spikes but not up to the initial values. The patient received clindamycin for 46 days and IV vancomycin for 28 days. She had an improved fever curve but continued with intermittent temperature spikes, averaging one per day between 38.0 ℃ and 38.5 ℃. There was concern for possible drug fever as a cause of the spike for which vancomycin was stopped. She was switched to linezolid on day 47 and discharged home on oral linezolid to continue until CRP normalizes.

**Figure 5:**
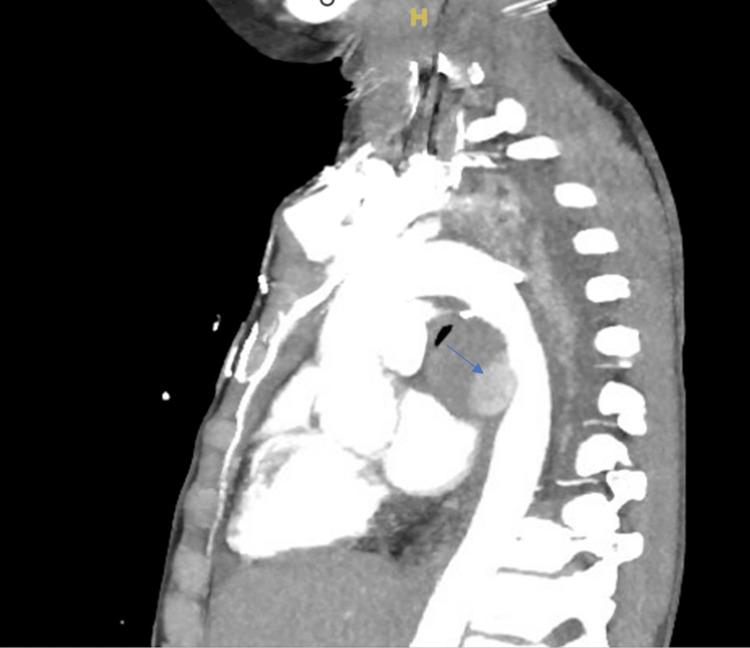
Computed tomography of the chest with contrast The blue arrow shows a pseudoaneurysm of the descending aorta.

However, the patient was readmitted four days after discharge for complaints of left-sided back pain and difficulty breathing. She was hypoxic, tachycardic, and hypertensive. On admission, CXR was suggestive of pneumonia, atelectasis, and mucus plug. Transthoracic echocardiography showed slight thickening of the mitral valve leaflets with borderline to mild anterior leaflet prolapse resulting in mild regurgitation. Biventricular cavity size, wall thickness, and systolic function were normal, with no evidence of pulmonary hypertension or pericardial effusion. The CT chest with contrast showed a pseudoaneurysm of the descending aorta and larger fluid collection, causing possible external compression on the left mainstem bronchus (Figure 5).

**Figure 6:**
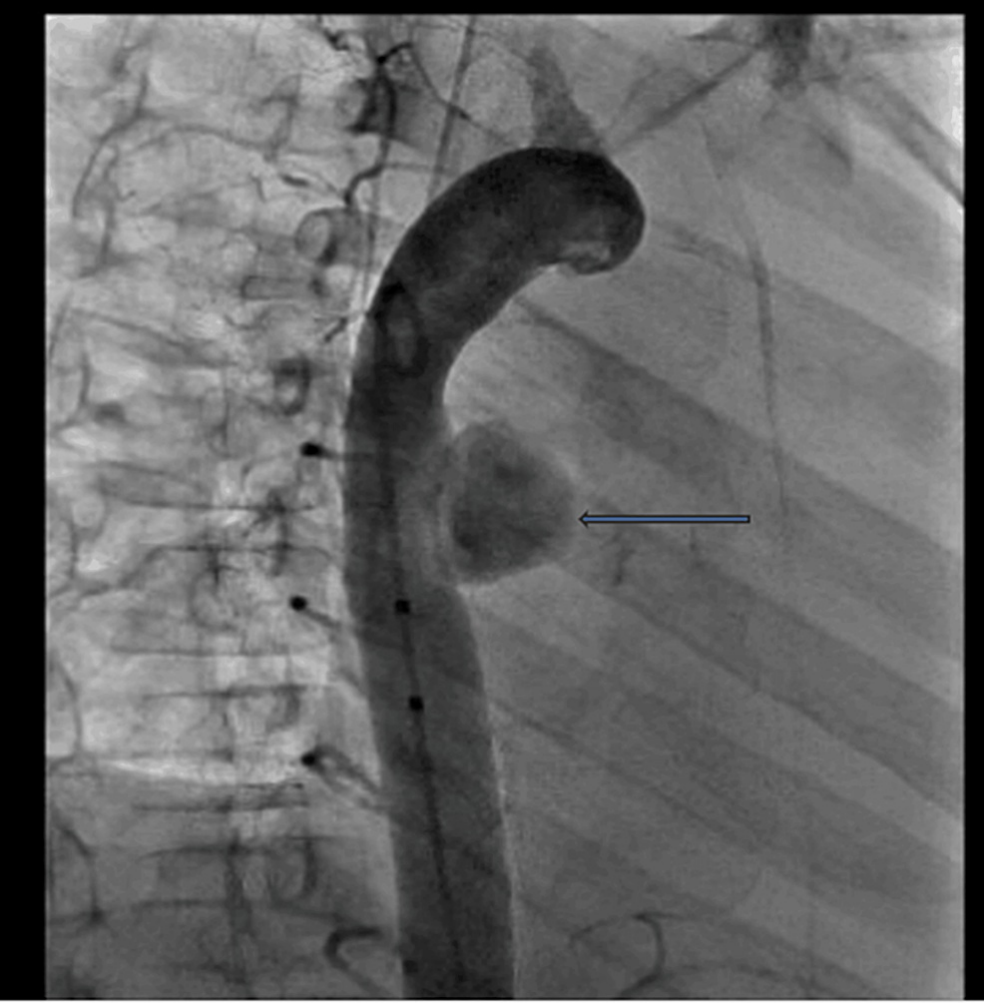
Cardiac angiography of the heart The blue arrow shows a pseudoaneurysm of the descending aorta.

She had a cardiac catheterization and angiography, which showed a large aortic aneurysm (Figure 6) for which a coil occlusion was done.

She was started on anti-hypertensive medications and discharged to stay locally for close monitoring while continuing her linezolid medication.

## Discussion

During the early days of the current CA-MRSA epidemic, CA-MRSA was mostly seen and associated with patients with predisposing risk factors. However, as the years went by, there was an increase in infection among children without predisposing risk factors [9]. Our patient had no known congenital disease, Intravenous illicit drug use, or immunological disorder.

SCC mec type IV has been reported to be the most common type seen in the US, Europe, and Australia [10]. Panton-Valentine leucocidin (PVL) toxin has been credited as a major virulent factor in complicated cases of CA-MRSA [11] but has also been reported absent in most CA-MRSA [12].

MRSA septicemia leads to widespread systemic effects. Our patient developed endocarditis during her first admission, which resolved with appropriate IV antibiotic therapy; however, was readmitted with severe chest pain and found to have a pseudoaneurysm. A thoracic aortic aneurysm is rare and is associated with a high mortality rate in children and adolescents [13]. Naidu DP et al. reported a case of mycotic aneurysm after a CA-MRSA infection in an adolescent female in Texas who presented with chest pain and fever similar to our patient [13]. Unlike our patient, another case reported in a child occurred post-catheterization (predisposing risk factor) [14]. Due to our patient’s body habitus, echocardiography, the first line of investigation [14], did not provide a diagnosis. A chest CT helped identify the pseudoaneurysm.

Psoas abscess and osseous infection are other common complications of CA-MRSA septicemia. Most reported cases with good outcomes had surgical and antibiotic therapy, as in our patient. A psoas abscess can be primary in origin, commonly seen in children, or secondary from adjacent structures. In our patient who had osteomyelitis of the hip with two repeat psoas abscesses, it is difficult to differentiate if it was from the hip bone infection or due to her fall (history of trauma). It is also difficult to state if the fall was the point of MRSA inoculation from the hip joint. In our patient, multiple surgical interventions and prolonged antibiotics resulted in a fair outcome.

Necrotizing pneumonia is an uncommon complication of CA-MRSA pneumonia and has been on the rise since the early 1990s [15]. The diagnosis is often made with CT chest imaging showing loss of pulmonary architecture, decreased parenchymal enhancement, and multiple thin-walled cavities, similar to the CT imaging in our patients [15]. There are controversial reports on the role of PVL in invasive diseases like pneumonia. While some studies report PVL as a major virulence factor for necrotizing pneumonia [11], other studies have refuted this claim [15,16]. In our patient, PVL virulence factor marker testing was not done.

The recommended treatment for CA-MRSA bacteremia with infective endocarditis is intravenous vancomycin, usually of two to six weeks duration [17]. Our patient received a long duration of IV clindamycin before switching to oral linezolid. Vancomycin was used for about 28 days and discontinued for concerns of drug fever as a cause of the intermittent fever spike on admission after achieving a downtrending CRP. Linezolid was continued post-discharge and on readmission.

## Conclusions

In conclusion, CA-MRSA is a cause of morbidity and mortality in children. Disseminated infection with multisystem involvement should be suspected in children without predisposing risk factors. Both medical and surgical management are necessary for a favorable outcome. Since cardiac complications from MRSA infection, such as aortic aneurysms, have been reported in the literature, close outpatient monitoring with echocardiography should be considered in patients managed for disseminated infection to avoid mortality.
